# Convergent Validity of the Arab Teens Lifestyle Study (ATLS) Physical Activity Questionnaire

**DOI:** 10.3390/ijerph8093810

**Published:** 2011-09-23

**Authors:** Hazzaa M. Al-Hazzaa, Hana I. Al-Sobayel, Abdulrahman O. Musaiger

**Affiliations:** 1 Exercise Physiology Laboratory, Department of Physical Education and Movement Sciences, College of Education, King Saud University, P.O. Box 2458, Riyadh, 11451, Saudi Arabia; 2 Scientific Board Member, Obesity Research Chair, King Saud University, Riyadh, Saudi Arabia; 3 Department of Rehabilitation Sciences, College of Applied Medical Sciences, King Saud University, P.O. Box 6941, Riyadh, 11452, Saudi Arabia; E-Mail: hsobayel@ksu.edu.sa; 4 Arab Center for Nutrition, Manama, Bahrain, and Nutrition and Health Studies Unit, Deanship of Scientific Research, University of Bahrain, P.O. Box 26923, Manama, Bahrain; E-Mail: amusaiger@gmail.com

**Keywords:** Arab Teens Lifestyle Study, validity, questionnaire, pedometer

## Abstract

The Arab Teens Lifestyle Study (ATLS) is a multicenter project for assessing the lifestyle habits of Arab adolescents. This study reports on the convergent validity of the physical activity questionnaire used in ATLS against an electronic pedometer. Participants were 39 males and 36 females randomly selected from secondary schools, with a mean age of 16.1 ± 1.1 years. ATLS self-reported questionnaire was validated against the electronic pedometer for three consecutive weekdays. Mean steps counts were 6,866 ± 3,854 steps/day with no significant gender difference observed. Questionnaire results showed no significant gender differences in time spent on total or moderate-intensity activities. However, males spent significantly more time than females on vigorous-intensity activity. The correlation of steps counts with total time spent on all activities by the questionnaire was 0.369. Relationship of steps counts was higher with vigorous-intensity (r = 0.338) than with moderate-intensity activity (r = 0.265). Pedometer steps counts showed higher correlations with time spent on walking (r = 0.350) and jogging (r = 0.383) than with the time spent on other activities. Active participants, based on pedometer assessment, were also most active by the questionnaire. It appears that ATLS questionnaire is a valid instrument for assessing habitual physical activity among Arab adolescents.

## 1. Background

According to the World Health Organization, the most important risk factors of non-communicable diseases in the Arab countries include high blood pressure, high concentrations of cholesterol in the blood, inadequate intake of fruit and vegetables, overweight or obesity, physical inactivity and tobacco use [1]. Five of these risks are closely related to improper diet and physical inactivity. It is believed that reducing the burden of noncommunicable diseases depends on controlling several modifiable risk factors, including physical inactivity [2]. Indeed, physical inactivity is considered a major risk factor for a number of chronic diseases, including cardiovascular diseases, diabetes mellitus, obesity, osteoporosis and certain types of cancer [2–4]. On the other hand, physical activity in young people is a key determinant of energy expenditure and leads to improved cardiovascular and metabolic fitness as well as enhanced bone health [5]. As a result, monitoring physical activity levels in the society is increasingly becoming a public health priority [4,6].

It is well recognized that accurate assessment of physical activity levels is an important factor for understanding the association between active lifestyle and health. In addition, precise measurement of physical activity is crucial when monitoring secular trends or evaluating the effectiveness of intervention programs [6]. A variety of physical activity measurement methods exist. Each one of these methods has its own advantages and disadvantages. The choice of an instrument depends on its purpose, characteristics of the target population and available resources. Nevertheless, the use of self-reported questionnaire in population studies is the most common method for physical activity assessment [7]. It is easy to administer, more economical and provides important information on the type and domains of physical activity that is not possible by an objective measure [8].

However, before using a self-reported questionnaire for physical activity assessment, an acceptable validity coefficient for such an instrument need to established. A recent systematic review concluded that there is no physical activity questionnaire that has both acceptable reliability and validity for youth [9]. Furthermore, in the Arab population, instruments that have been developed and validated for the assessment of physical activity among young people are very scarce [10,11]. Previous research has also indicated that reliability and validity of physical activity questionnaire varied by race [12]. Therefore, it was the purpose of the present paper to report on the convergent validity of the Arab Teens Lifestyle Study (ATLS) physical activity questionnaire against an electronic pedometer. The ATLS is a multicenter collaborative research project for the assessment of physical activity and other lifestyle habits of adolescents in selected major Arab cities [13]. We hypothesized that the relationships between the ATLS physical activity questionnaire and pedometer readings would be the highest during ambulatory activities such as walking and running and the lowest during non-ambulatory activities such as bicycling, swimming and weight training.

## 2. Methods

### 2.1. Study Sample

A subsample of 36 females (age: 16.3 ± 1.2 years) and 39 males (age: 16.0 ± 0.9 years), who were randomly selected from an ATLS sample at Riyadh city [13] in Saudi Arabia (total sample was 1001 participants), represented the validation study. The participants were drawn from two classes selected randomly from each of the chosen female and male schools. The original total sample of ATLS was selected using a multistage stratified sampling technique. At the first stage, a systematic random sampling procedure was used to select the schools. The schools were stratified into boys and girls secondary schools, with further stratification into public and private schools. There were no significant differences (*p* < 0.05) in age, height, weight or body mass index between the present validation sample and the original sample of ATLS from Riyadh. The study protocol was approved by the Research Center at King Saud University as well as by the General Directorate of School Education and schools principles in Riyadh. Participants and their parents were all informed of the study procedures and were given written instruction on how to keep the pedometers in place during the study period. Body weight was measured without shoes and with minimal clothing to the nearest 0.1 kg using a Seca digital scale (model 770, Seca, Germany). Standing height was measured to the nearest 0.1 cm without shoes using a calibrated measuring rod (Seca Road Rod portable stadometer, Seca, Germany).

### 2.2. Physical Activity Assessment

The ATLS physical activity questionnaire [14] is a modified questionnaire based on a previously developed physical activity questionnaire for adolescents and young adults. The original questionnaire was previously shown to have a high reliability (ICC = 0.85; 95% CL = 0.70–0.93) and acceptable validity (r = 0.30; p < 0.05) against pedometer using a convenient sample of males 15–25 year-old [10,11]. However, the previous validation study was conducted on males only and with a convenient sample with a wide age range (15–25 years). The ATLS project was a recent initiative to assess the physical activity patterns, sedentary activity and eating habits of randomly selected samples of secondary-school boys and girls living in major Arab cities [13]. The participating students from Riyadh sample completed the questionnaire using the Arabic version while in their classrooms with the supervision of their teachers and in front of at least one of the research assistants. The questionnaire was designed to collect information on frequency, duration and intensity of variety of light-, moderate- and vigorous-intensity physical activities during a typical (usual) week. The physical activity questionnaire covers such domains as transport, household, fitness and sports activities. Moderate-intensity physical activity includes activities such as brisk walking, recreational swimming, household activities and moderate-intensity recreational sports like volleyball, badminton, table tennis and a like. They were assigned metabolic equivalent (MET) values equivalent to 3–6 METs based on the compendium of physical activity [15] and the compendium of physical activity for youth [16]. Vigorous-intensity physical activity requires energy expenditure greater than six METs, and includes activities such as stair claiming, jogging, running, cycling, self-defense, weight training and vigorous-intensity sports such as soccer, basketball, handball, single tennis and a like. Slow walking, normal pace walking, and brisk walking were assigned MET values of 2.8, 3.5 and 4.5 METs, respectively, based on a modified MET values from the compendium of physical activity for youth [16]. Household activities were given the average MET value of 3. This because they include some items that may require less than three METs like washing dishes (2.5 METs), cleaning bathroom (2.5 METs), cooking (2.5 METs), and ironing (2.3 METS) as well as other household activities that require three METs or above like car washing (3 METs), vacuuming (3.5 METs), mopping (3.5 METs) and gardening (3.5 METs). To measure the participants’ levels of physical activity, we used the total METs-min per week and the METs-min per week spent in each of the moderate- and vigorous-intensity physical activity.

Following questionnaire administration, objective measurement of physical activity was assessed using electronic pedometer (Digi-walker SW 701, Tokyo, Japan) for three continuous weekdays. A previous report [17] has indicated that three days measurements provide a reliable estimate of daily step counts, with intraclass correlation coefficient of 0.80. Furthermore, Craig *et al*. [18] assessed a large sample of Canadian youth using pedometers and found a reliability of more than 90% for the three day-measurement. Pedometers are simple and inexpensive body-worn motion sensors that are increasingly used for objective assessment of physical activity behaviors. Studies have showed that pedometer counts to correlate strongly (r = 0.86) with accelerometry [19]. The type of pedometer used in the present study is found to be valid for measuring steps [20]. The instrument has been previously used on a similar population and does not interfere with the participant’s normal daily activity [21]. The pedometer was securely attached to the right hip of the participant at the beginning of school day, and right after the participant has finished answering the questionnaire forms. Complete instructions were given to the participants to keep the pedometer in place all the time except when showering, swimming and sleeping. The pedometers were sealed and were collected from the participants after three days at the same time when they were placed. Eighty five pedometers were placed on the selected students. However, only 75 pedometers had complete data. The rest of pedometers had either incomplete data or were lost.

### 2.3. Data Analysis

Data entry and statistical analysis were performed using the Statistical Package for the Social Science (SPSS) program, version 15 (SPSS, Inc., Chicago, IL, USA). To avoid over-reporting, physical activity scores were cleaned and truncated at reasonable and realistic levels, taking into account the fairly long time spent on learning at schools, doing homework, sleeping and TV viewing and computer use time. The maximal total time spent on physical activity per week was truncated at 1,680 minutes (28 hours), or four hours of physical activity per day. Results are expressed as means and standard deviations or percentages. Since the variable total minutes spent in physical activity from the questionnaire output was not normally distributed, log transformation was performed to the data before applying the correlation analysis. Pearson’s correlation coefficient was used to determine the relationships between pedometer steps counts and time spent during total, moderate-, and vigorous-intensity physical activities. In addition, relationships between pedometer counts and time spent during specific activity such as walking, jogging, bicycling, swimming, weight training and household activities were examined to check for the differences in the correlation coefficients between ambulated and non-ambulated types of movement. Partial correlation while controlling for gender was also used. Finally, independent t-test was used to test mean differences in pedometer steps counts and total activity time (by questionnaire) between the active and inactive participants, based on the upper and lower 50% percentiles of pedometer-determined step counts.

## 3. Results

Descriptive characteristics of the sample are shown in Table 1. Males were significantly taller and heavier than females (*p* < 0.01), whereas mean values for body mass index (BMI) were not significantly different between males and females. The findings showed a wide variation of BMI among participants, ranging from 15.1 to 43.4 kg/m^2^. There was no significant gender difference observed for total steps counts per day. There were only two males and two females who exceeded the cut-off daily pedometer counts of 15,000 and 12,000 steps for males and females, respectively [22]. However, there were more males than females who achieved an absolute cut-score of 10,000 or more steps per day. Furthermore, based on questionnaire results, there was no significant gender difference in total time spent on all types of physical activities or on moderate-intensity activities. However, males spent significantly more time than females engaging on vigorous-intensity physical activities (*p* < 0.001). The proportion of participants who achieved 150 minutes or more per week engaging on all physical activities, based on the questionnaire results, were 66.7% for males and 52.8% for females. However, less than half the males (46.2%) and only one third (33.3%) of the females achieved 300 minutes or more per week engaging in physical activity.

In addition, the proportions of males *versus* females who reported no walking, slow walking, normal pace walking and brisk walking were 12.8% *versus* 38.9%, 12.8% *versus* 8.3%, 66.7% *versus* 52.8% and 7.7% *versus* 0.0%, respectively (Chi-square test was significant at 0.033 level).

Table 2 presents the correlation coefficients findings of pedometer steps counts with questionnaire-based physical activity indicators. The correlation of steps counts with total time spent on all activities by the questionnaire was 0.369 (*p* = 0.001). The strength of relationship did not seem to be greatly affected by gender, since the partial correlation while controlling for gender dropped only slightly (r = 0.349; *p* = 0.002). The coefficient of relationships of steps counts was much higher with vigorous-intensity physical activity (r = 0.338; *p* = 0.003) than with moderate-intensity activity (r = 0.265; *p* = 0.022). In addition, pedometer steps counts showed higher correlations with time spent on walking (r = 0.350; *p* = 0.002) and jogging (r = 0.383; *p* = 0.001) than with the time spent on swimming (r = 0.137; *p* = 0.243), household activity (r = 0.135; *p* = 0.247), bicycling (r = 0.115; *p* = 0.324), martial arts (r = 0.102; *p* = 0.384) or weight training (r = 0.042; *p* = 0.721). When the correlation between steps counts and total energy expenditure in MET-minutes per week was calculated, the coefficient of relationship has slightly increased (r = 0.371; *p* = 0.001).

Table 3 shows the results of grouping the sample into the most and the least active groups, based on the upper and lower 50th percentiles of pedometer-determined steps counts. From the table, it is clear how the figures of the steps counts and the time spent on physical activity by the questionnaire are consistent among the more active and the less active groups. It appears that those participants who were most active based on pedometer assessment spent also significantly more time engaging in physical activity based on questionnaire findings, though there were no significant differences between the two groups in age or BMI values. Moreover, there were no significant gender differences in total activity time whether below (*p* = 0.375) or above (*p* = 0.785) the 50th percentiles of pedometer-determined steps counts.

## 4. Discussion

The present study found fair and significant validity coefficients between pedometer-determined steps counts and total time spent on activity based on ATLS physical activity questionnaire. The associations were higher with ambulatory activities such as walking, jogging and running compared to non-ambulatory activities such as bicycling, martial arts, weight training and some household activities. Gender differences did not greatly affect the strength of relationship between steps counts and total time spent in physical activity. The pedometer that was utilized as a convergent measure in the current study has been extensively used in previous reports and showed a fair validity and a high reliability [19].

The strength of the association observed in the present validity study is similar to the upper range of correlations that have been previously shown between self-reported physical activity questionnaires and objectively assessed activity levels in adult population [23]. In another review paper [19], the median correlation of pedometer counts with self-reported physical activity questionnaire was shown to be 0.33; a value that is very close in magnitude to what has been found in the present study. A similar correlation (r = 0.33–0.35) was also found between Bouchard Activity Diary and ActiGraph activity monitor in Spanish adolescents [24]. Another validity study on Vietnamese adolescents reported an acceptable coefficient (r = 0.27) when comparing physical activity questionnaire with accelerometer measurement [25]. Lower validity coefficients (r = 0.17–0.30) were found for the modified version of International Physical Activity Questionnaire in European adolescents [26], as well as for the activity questionnaire for adults and adolescents (AQuAA) from The Netherlands [27]. A recent systematic review on the validity of physical activity questionnaires for young people in the European countries reported a fair validity for the majority of these instruments [28]. However, a somewhat higher correlation (r = 0.45) between physical activity questionnaire for Swedish adolescents and accelerometer measurement was reported elsewhere [29].

In the present study, the correlation between pedometer steps counts and ATLS questionnaire was higher for the time spent on vigorous-intensity physical activity compared to moderate-intensity activity. The higher correlations with activity such as jogging and running may have greatly contributed to the strength of the association. In addition, jogging and running involve frequent stepping, activity that pedometer is built to accurately capture and record. In addition, the planned nature of the vigorous physical activity perhaps makes them easier to be recalled. Our hypothesis that the relationships between pedometer counts and the activity questionnaire would be the highest in ambulatory activity and the lowest in the non-ambulatory activity was fulfilled. Pedometer usually records ambulatory activities and may not capture all other types of physical activity. It is also worth noting that in adult studies validity coefficient for household activities tended to be lower [30], something that is similar to what has been found in the present study.

Based on the results of the pedometer step counts reported in the present study, it appears that a large proportion of the participants were generally not active enough. Only one third of the males and about 14% of the females had pedometer count above a cut score of 10,000 steps per day. A recent study using pedometer counts suggested a zone-based hierarchy for measurement and motivational purposes in adults. The suggested cut-off range for the low active category was 5,000–7,499 steps per day [31]. Applying such cut-off scores to our data resulted in having more than half of the males and about three quarter of the females were being classified as insufficiently active. Moreover, studies have shown that boys report more moderate-to vigorous intensity physical activity than girls [32,33]. In our questionnaire findings, males reported significantly more vigorous-, but not moderate-intensity physical activity compared to females. However, the intended purpose of present study was not to focus on the gender differences in physical activity prevalence.

Comparison made between questionnaires and objective measures of physical activity such as activity monitors may under predict the questionnaire capacity to provide an accurate estimate of total physical activity. Thus, a high correlation between the questionnaire result and the motion sensor outcome should not be expected, since both measures intend to assess differing components of the activity behavior [23]. Finally, the assessment of physical activity by the questionnaire method, though has many advantages, suffers from several limitations that must be kept in mind. These limitations include the social desirability effect of the questionnaire and the recall bias. In addition, respondents may sometimes have difficulty understanding obscure terms such as physical activity, moderate-intensity and a like. We have validated ATLS questionnaire against electronic pedometer, and although pedometer is a reliable instrument and provides an objective estimate of the level of physical activity, it is of course not the gold standard criterion for assessing habitual physical activity [7,19]. Thus, pedometer, in the present study, could have underestimated the actual physical activity levels of the participants. Pedometry is not sensitive to activities like swimming, bicycling, martial arts, weight training and most household activities. This was evident by the lower correlations of pedometer steps counts with time spent on these activities that were reported in our study. Furthermore, pedometer is not capable of assessing the intensity of physical activity, as the correlation of pedometer output did not improve much when the total physical activity was calculated as MET-min per week instead of only minutes per week. The current study examined the relationship between pedometer counts and questionnaire results using Person correlation, but no absolute agreement was provided between the two instruments. Moreover, in the present study, we only collected step counts for three week days. It would have been much better had we placed the pedometer on the participants for one week, so to include weekend days too. However, that would have required more time and a much larger number of pedometers than what we had.

We can conclude that the findings of the present study provide support for the validity of the ATLS questionnaire as an instrument for the assessment of habitual physical activity among Arab adolescents with varying body mass index and irrespective of gender type. The ATLS physical activity questionnaire was designed to collect complete information on frequency, duration and intensity of light-, moderate- and vigorous-intensity physical activities, which makes it a robust instrument for capturing all youth physical activities related to transport, household, fitness and sports domains.

## Figures and Tables

**Table 1:** Means and standard deviations of descriptive characteristics of the participants (n = 75).

Variable	Males (n = 39)	Females (n = 36)	*P* value
Age (year)	16.0 ± 0.9	16.3 ± 1.2	0.170
Weight (kg)	67.4 ± 17.4	57.8 ± 14.3	0.010
Height (cm)	168.5 ± 6.0	155.2 ± 5.3	0.000
Body Mass Index	22.8 ± 6.3	23.9 ± 5.5	0.910
Pedometer steps counts (steps/day)	7,514 ± 4,106	6,164 ± 3,482	0.131
Percent achieving ≥ 10,000 steps/day (%)	33.3	13.9	-
Percent achieving ≥ 7,500 steps/day (%)	46.2	27.8	-
Percent achieving ≥ 5,000 steps/day (%)	66.7	38.9	-
*Questionnaire (min/week)*:			
Total time spent in all activities 1	471.8 ± 442.2	358.5 ± 426.5	0.264
Time spent on moderate-intensity activities	199.2 ± 260.8	267.0 ± 323.9	0.319
Time spent on vigorous-intensity activities	267.9 ± 273.6	89.4 ± 143.6	0.001
Percent achieving ≥ 150 min/week (%) 2	66.7	52.8	-
Percent achieving ≥ 300 min/week (%) 3	46.2	33.3	-

*P* value = probability level for testing the differences between males and females.

1 The total time spent on all activity is the sum of time spent on vigorous-intensity, moderate-intensity, and light-intensity physical activity (mainly slow walking);

2 An equivalent of 30 min per day, five times per week;

3 An equivalent of 60 min per day, five times per week.

**Table 2:** Pearson correlation coefficients (r) of pedometer steps count per day with the time spent (min/week) on physical activity using ATLS questionnaire for the whole group.

Variable	r	*P* value
Total time spent on all activities	0.369	0.001
Time spent on moderate-intensity activities	0.265	0.022
Time spent on vigorous-intensity activities	0.338	0.003
*Time spent on specific activity:*		
Time spent on walking	0.350	0.002
Time spent on jogging	0.383	0.001
Time spent on swimming	0.137	0.243
Time spent on house-hold activities	0.135	0.247
Time spent on bicycling	0.115	0.324
Time spent on martial arts	0.102	0.384
Time spent on weight training	0.042	0.721

**Table 3:** Mean and standard deviation of pedometer-determined steps counts and total activity time spent on physical activities using ATLS questionnaire, according to values above and below the 50th percentiles of pedometer steps counts.

Variable	Pedometer steps counts percentiles		*P* value
	<50th	>50th	
Age (years)	16.3 ± 1.3	15.9 ± 0.94	0.079

Body mass index (kg/m^2^)	23.6 ± 5.7	24.0 ± 6.1	0.733

Pedometer steps counts (steps/day)	3,778 ± 1,718	9,873 ± 2,831	0.000
Total activity time (min/week)	284.1 ± 312.0	547.2 ± 499.6	0.008
